# Physiological Persistence of Tension Pneumothorax After Minor Diaphragmatic Injury During Laparoscopic Adrenalectomy: A Case Report

**DOI:** 10.1155/cria/6594164

**Published:** 2026-04-27

**Authors:** Eun Ji Park, Jeong-Min Hong, Hyeon-Jeong Lee, Unji Kim, Wangseok Do

**Affiliations:** ^1^ Department of Anesthesia and Pain Medicine, Medical Research Institute, Pusan National University Hospital, Busan, South Korea, mri.gov.lk; ^2^ Department of Anesthesia and Pain Medicine, School of Medicine, Pusan National University, Yangsan, South Korea, pusan.ac.kr

**Keywords:** diaphragmatic injury, general anesthesia, laparoscopy, physiological recovery, pneumoretroperitoneum, tension pneumothorax

## Abstract

**Background:**

During laparoscopic surgery, tension pneumothorax may persist despite prompt anatomical repair of a diaphragmatic injury, posing a diagnostic and management challenge for anesthesiologists under general anesthesia.

**Case:**

A 68‐year‐old male undergoing bilateral laparoscopic adrenalectomy using a retroperitoneal approach developed progressive hypoxemia, hypercapnia, elevated peak inspiratory pressure, and hemodynamic instability approximately 4 h after surgical initiation. A minor diaphragmatic injury was identified and immediately repaired after reduction of pneumoretroperitoneum. Despite anatomical correction, respiratory and circulatory instability persisted, requiring high‐dose vasopressor support and 100% inspired oxygen until the end of surgery. A radiograph obtained at the conclusion of surgery demonstrated marked mediastinal shift consistent with tension pneumothorax. The pneumothorax resolved spontaneously with supportive ventilation, and the patient recovered without chest tube insertion.

**Conclusion:**

This case highlights a physiological pitfall rather than a rare complication: even a minor diaphragmatic injury can result in sustained tension physiology despite timely anatomical repair during prolonged laparoscopic surgery. Continuous vigilance for evolving physiological abnormalities and proactive anesthesiologist‐led management are essential.

## 1. Introduction

Laparoscopic surgery is widely adopted for abdominal procedures because of its advantages over open surgery, including reduced postoperative pain, shorter hospital stay, and faster recovery [1]. Creation of pneumoretroperitoneum using carbon dioxide is fundamental to laparoscopic techniques; however, it may also lead to characteristic cardiopulmonary complications, particularly when surgical manipulation involves the diaphragm.

Tension pneumothorax is a potentially life‐threatening condition that can occur during laparoscopic surgery when retroperitoneal gas migrates into the thoracic cavity through a diaphragmatic defect [2–4]. Under general anesthesia, diagnosis is especially challenging because classical symptoms such as chest pain and dyspnea are absent, and early manifestations are often subtle and nonspecific [5, 6]. Consequently, anesthesiologists must rely primarily on indirect physiological indicators, including progressive hypoxemia, rising airway pressures, reduced tidal volume (TV) delivery, and increasing end‐tidal carbon dioxide (EtCO_2_) levels.

Diaphragmatic injuries encountered during laparoscopic procedures are frequently small and may appear clinically insignificant at the time of detection [7]. Nevertheless, even minor defects can serve as occult pathways for retroperitoneal gas migration under sustained pneumoretroperitoneum, resulting in tension physiology and hemodynamic compromise. Importantly, anatomical repair of a diaphragmatic injury does not always result in immediate physiological recovery.

Here, we report a case of intraoperative tension pneumothorax following a minor diaphragmatic injury during bilateral laparoscopic adrenalectomy. This case illustrates a critical physiological pitfall in which cardiopulmonary instability persisted despite timely anatomical correction, underscoring the importance of continuous intraoperative vigilance and anesthesiologist‐led management.

## 2. Case Report

A 68‐year‐old male patient (height: 166 cm and weight: 55 kg) was scheduled to undergo bilateral laparoscopic adrenalectomy for adrenal carcinoma. The procedure was performed using a retroperitoneal approach in the prone position. The patient had a history of acute myocardial infarction treated with percutaneous coronary intervention and stent placement 3 years prior to presentation. Preoperative transthoracic echocardiography demonstrated preserved left ventricular systolic function (ejection fraction 53%) with regional wall motion abnormality in the basal inferior segment, consistent with prior ischemic insult, as well as diastolic dysfunction. Mild tricuspid regurgitation and left atrial enlargement were also noted. Pulmonary function tests revealed a moderate obstructive pattern (FEV1/FVC 53%), consistent with underlying chronic obstructive pulmonary disease. The patient was receiving standard medical therapy for ischemic heart disease, including antiplatelet and statin medications. Overall, these findings suggested limited cardiopulmonary reserve, which may have influenced the patient’s physiological response to intraoperative stress. Routine laboratory tests, chest radiography, and electrocardiography revealed no significant abnormalities.

Upon arrival in the operating room, the patient’s vital signs were as follows: blood pressure, 168/99 mmHg; heart rate, 103 beats per minute; and peripheral oxygen saturation (SpO_2_), 95%. After preoxygenation with 100% oxygen, general anesthesia was induced with intravenous propofol (130 mg) and rocuronium (50 mg), and a continuous infusion of remifentanil was initiated at 0.5 mg/h. Endotracheal intubation was performed using a video laryngoscope, and bilateral breathing sounds were confirmed on auscultation. Anesthesia was maintained with desflurane (6.0 vol%) in 40% oxygen and continuous remifentanil infusion. The patient was positioned prone for surgery. Mechanical ventilation was initiated in volume‐controlled mode with a TV 450 mL, respiratory rate (RR) 16 breaths per minute, and positive end‐expiratory pressure (PEEP) 5 cmH_2_O. EtCO_2_ was 34–35 mmHg, peak inspiratory pressure (PIP) 18–20 cmH_2_O, and SpO_2_ 100%.

Approximately 4 h after the commencement of surgery, a progressive decline in SpO_2_ to 80% was observed, accompanied by elevations in EtCO_2_ to 50 mmHg and PIP to 40 cmH_2_O, along with inadequate TV delivery. At that time, blood pressure was 106/72 mmHg, and heart rate was 105 beats per minute. Manual ventilation with 100% oxygen was initiated immediately, resulting in partial improvement in oxygen saturation to 90%. Arterial blood gas analysis (ABGA) showed pH 7.153, partial pressure of arterial carbon dioxide (PaCO_2_) 85.5 mmHg, partial pressure of arterial oxygen (PaO_2_) 82.1 mmHg, and oxygen saturation 91.2%. Five minutes later, hypotension (85/61 mmHg) and tachycardia (121 beats per minute) developed, prompting the initiation of norepinephrine infusion.

At the anesthesiologist’s request for minimal pneumoretroperitoneum, the surgeon identified a minor defect in the left hemidiaphragm. Suspecting pneumothorax, the anesthesiologist alerted the surgeon, who requested consultation with thoracic surgeons. Ventilation was converted to pressure‐controlled mode with reduced TV and increased RR to maintain minute ventilation. Chest tube insertion was not performed because the defect appeared minor and was promptly repaired intraoperatively.

**FIGURE 1:**
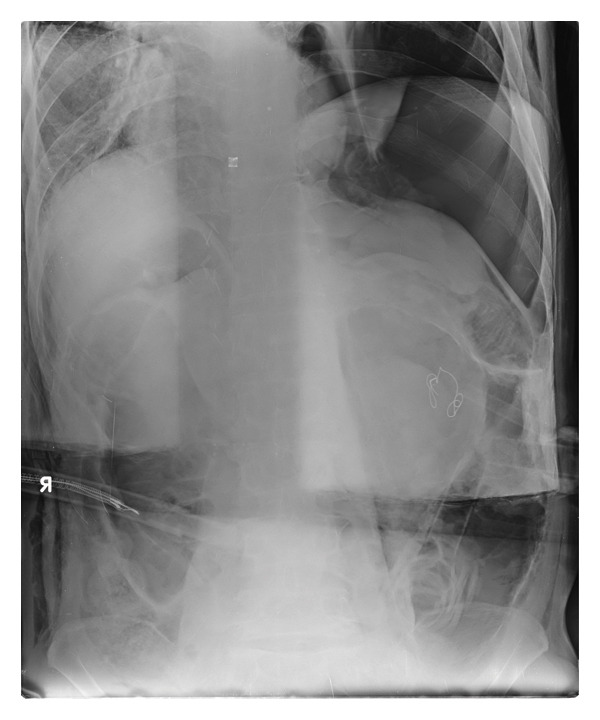
Portable abdominal radiograph obtained at the conclusion of surgery demonstrating a marked rightward mediastinal shift consistent with tension pneumothorax. Displacement of the heart and mediastinal structures suggests elevated intrathoracic pressure. The image was acquired in the prone position and horizontally inverted to maintain anatomical orientation.

Despite prompt repair of the diaphragmatic injury, 100% inspired oxygen was required until the conclusion of surgery. Hemodynamic instability persisted, requiring norepinephrine infusion up to 0.2 μg/kg/min to maintain systolic blood pressure (SBP) > 100 mmHg. Approximately 90 min after the onset of the event, SpO_2_ improved to 95%–96%, but ABGA showed persistent hypercapnia (PaCO_2_ 79.8 mmHg) and acidemia (pH 7.140) despite normalized oxygenation (PaO_2_ 103.2 mmHg and O_2_ saturation 95.2%). The surgery was completed roughly 3 h after the event. A portable abdominal radiograph obtained at the conclusion of surgery for gauze counting revealed a marked rightward mediastinal shift consistent with tension pneumothorax (Figure 1).

**FIGURE 2:**
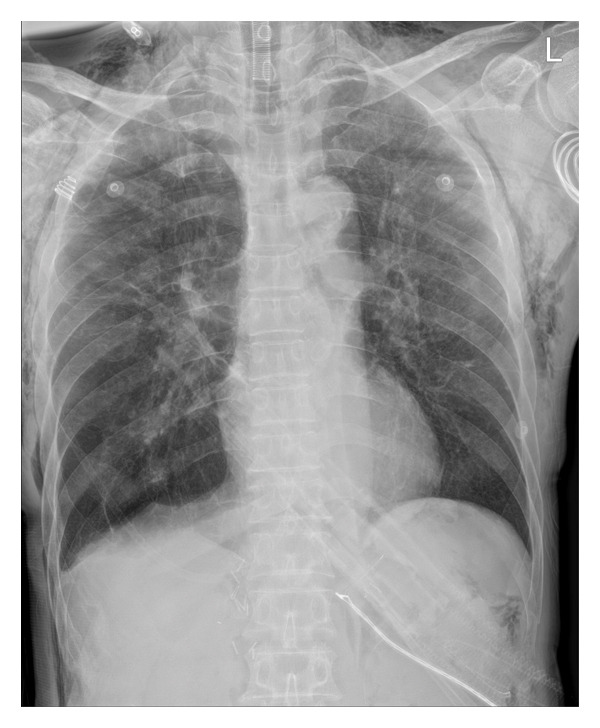
Portable chest radiograph obtained 1 h postoperatively showing spontaneous resolution of the pneumothorax. Lung expansion is restored, and the mediastinum has returned to the midline. The patient recovered without chest tube insertion.

The patient remained in the intensive care unit for observation and was managed with volume‐controlled ventilation (FiO_2_ 0.5, TV 400 mL, RR 16/min, and PEEP 5 cmH_2_O) to support spontaneous resolution of the pneumothorax through passive CO_2_ absorption, maintaining SpO_2_ at 100%. One hour postoperatively, chest radiography confirmed resolution (Figure 2). Norepinephrine, maintained at 0.1 μg/kg/min at the conclusion of surgery, was gradually tapered and discontinued. The following morning, sedation was discontinued, and the patient was extubated after demonstrating adequate spontaneous ventilation. He remained hemodynamically and respiratorily stable and was transferred to a general ward on postoperative day 1.

Intraoperative respiratory and hemodynamic parameters are summarized in Figure 3.

FIGURE 3Intraoperative trends in respiratory and hemodynamic parameters during bilateral laparoscopic adrenalectomy. (a) Respiratory parameters: oxygen saturation (SpO_2_), end‐tidal carbon dioxide (EtCO_2_), and peak inspiratory pressure (PIP). (b) Hemodynamic parameters: systolic blood pressure (SBP) and heart rate (HR). Four key intraoperative events are annotated: manual ventilation (305 min), initiation of norepinephrine infusion (310 min), diaphragm repair (345 min), and portable abdominal radiograph acquisition (420 min). Physiological deterioration preceded radiological confirmation and persisted despite anatomical repair.

**Figure:**
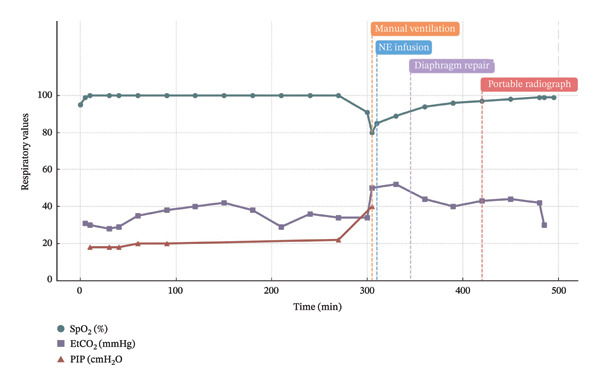
(a)

**Figure:**
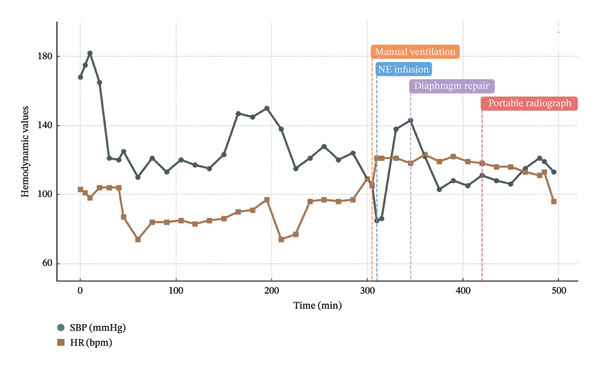
(b)

## 3. Discussion

Tension pneumothorax is an uncommon but potentially life‐threatening complication of laparoscopic surgery, particularly when the surgical field involves the diaphragm [2–4]. Under general anesthesia, its diagnosis is frequently delayed because classical symptoms such as chest pain and dyspnea are absent [5, 6]. Instead, anesthesiologists must rely on indirect physiological indicators, including progressive hypoxemia, rising airway pressures, reduced TV delivery, and increasing EtCO_2_ levels.

In the present case, these physiological abnormalities developed insidiously approximately 4 h after surgical initiation and progressed to severe hypercapnia, acidemia, and hemodynamic instability. Although a minor diaphragmatic injury was promptly identified and repaired, cardiopulmonary compromise persisted throughout the remainder of the operation. This temporal dissociation between anatomical correction and physiological recovery represents a critical intraoperative pitfall during prolonged laparoscopic procedures, as illustrated by the progressive changes in respiratory and hemodynamic parameters observed in this case (Figure 3).

**FIGURE 4:**
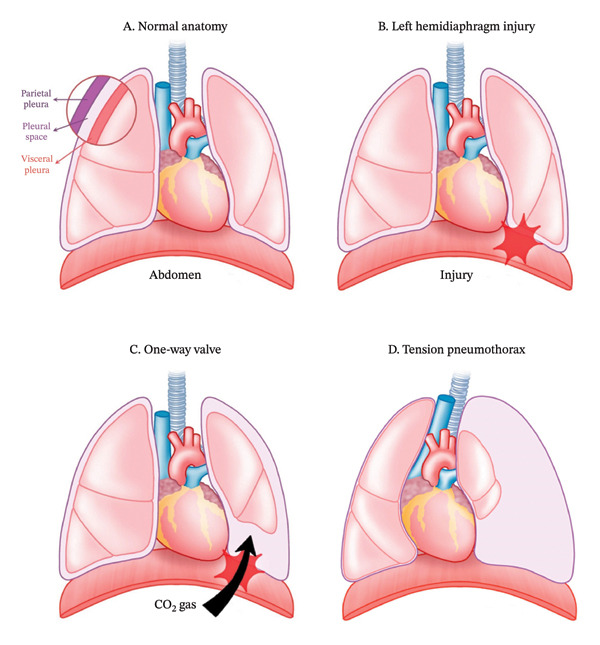
Schematic illustration summarizing the pathophysiological mechanism of tension pneumothorax associated with a minor diaphragmatic injury during laparoscopic surgery. (a) Normal thoracoabdominal anatomy, with the diaphragm separating the thoracic and abdominal cavities. (b) Development of a small defect in the left hemidiaphragm (indicated by a red star), creating potential communication between the retroperitoneal and pleural spaces. (c) Migration of retroperitoneal carbon dioxide through the defect under pneumoretroperitoneum, potentially functioning as a one‐way valve and allowing gas accumulation within the pleural cavity. (d) Accumulated intrathoracic gas leading to lung collapse, rightward mediastinal shift, impaired venous return, and hemodynamic instability consistent with tension physiology.

The underlying mechanism can be explained by the pressure gradient created by pneumoretroperitoneum, which may force carbon dioxide through even small diaphragmatic defects into the thoracic cavity [7]. When such a defect transiently functions as a one‐way valve, intrapleural pressure progressively increases, leading to lung collapse, mediastinal shift, impaired venous return, and reduced cardiac output—the hallmark features of tension physiology (Figure 4) [8]. Although carbon dioxide is highly soluble and may be absorbed once the source is controlled, this property does not mitigate the immediate hemodynamic consequences of tension pneumothorax.

Postoperative chest radiography is typically obtained after the resolution of pneumothorax and stabilization of cardiopulmonary status. As a result, the extent to which tension pneumothorax compresses the heart and contributes to intraoperative cardiovascular instability is rarely visualized. In most cases, anesthesiologists must infer the presence and severity of tension physiology based on evolving physiological deterioration rather than imaging findings. In the present case, radiological imaging was fortuitously obtained immediately before the conclusion of surgery, allowing visualization of a marked mediastinal shift consistent with ongoing tension pneumothorax. The marked mediastinal shift observed on the radiograph obtained at the conclusion of surgery (Figure 1) supports the physiological diagnosis of sustained tension pneumothorax made intraoperatively. Although imaging was not available at the onset of deterioration, the temporal relationship between physiological compromise and delayed radiological confirmation underscores the diagnostic value of continuous physiological monitoring when intraoperative imaging is not feasible.

Several perioperative factors likely amplified the physiological impact of tension pneumothorax in this patient, including surgical manipulation near the diaphragm [9], prolonged operative duration [10], prone positioning, and underlying chronic obstructive pulmonary disease with reduced pulmonary compliance [11]. These factors may obscure early clinical signs and exacerbate the cardiopulmonary consequences of intrathoracic gas accumulation.

Underlying cardiopulmonary comorbidities may have further reduced the patient’s physiological reserve, thereby amplifying the hemodynamic and respiratory consequences of tension pneumothorax. In particular, moderate obstructive lung disease and diastolic dysfunction may have impaired compensatory mechanisms during acute increases in intrathoracic pressure.

From a management perspective, anesthesiologist‐led interventions are essential. Immediate escalation of inspired oxygen concentration, manual ventilation, and appropriate ventilatory adjustments may stabilize gas exchange while definitive source control is achieved. During diaphragmatic repair, applying continuous positive airway pressure and evacuating pleural gas before final closure may reduce the transdiaphragmatic pressure gradient and limit further gas migration [12, 13]. Close interdisciplinary communication is equally critical to facilitate early recognition and timely intervention.

Although chest tube insertion is generally considered standard treatment for tension pneumothorax, conservative management may be appropriate in selected cases of carbon dioxide pneumothorax when the source is promptly controlled and physiological parameters stabilize [14, 15]. In the present case, subsequent radiography confirmed spontaneous resolution of the pneumothorax with supportive management alone (Figure 2). Nevertheless, early diagnosis and management should rely primarily on physiological deterioration rather than delayed imaging confirmation.

In summary, this case demonstrates that intraoperative tension pneumothorax should be regarded as a physiological diagnosis rather than solely an anatomical one. Even minor diaphragmatic injuries can precipitate sustained cardiopulmonary compromise under pneumoretroperitoneum despite timely repair. Continuous intraoperative vigilance, careful interpretation of physiological trends, and proactive anesthesiologist‐led management are crucial to preventing catastrophic outcomes during laparoscopic surgery.

## Funding

This work was supported by a 2‐Year Research Grant of Pusan National University.

## Ethics Statement

This case report was approved by the Institutional Review Board of Pusan National University Hospital (IRB no. 2509‐033‐155). Written informed consent for publication of this case report and accompanying images was obtained from the patient.

## Conflicts of Interest

The authors declare no conflicts of interest.

## Data Availability

The data that support the findings of this study are available on request from the corresponding author. The data are not publicly available due to privacy or ethical restrictions.
